# Cohort profile: the Korean National Health Examination Baseline (KNHEB) cohort for longitudinal health monitoring in South Korea

**DOI:** 10.1186/s12889-025-22856-4

**Published:** 2025-05-14

**Authors:** Suyoung Jo, Eunsil Cheon, Heewon Kang, Min Kyung Lim, Wankyo Chung, Sun Ha Jee, Keum Ji Jung, Yeun Soo Yang, Seong Yong Park, Sunmi Lee, Jin-Kyoung Oh, Kyoungin Na, Soyeon Kim, Jieun Hwang, Sung-il Cho

**Affiliations:** 1 Institute of Health and Environment, Seoul National University, Seoul, Republic of Korea; 2 Department of Public Health Science, Graduate School of Public Health, Seoul National University, 1 Gwanak-ro, Gwanak-gu, Seoul, 08826 Republic of Korea; 3 Department of Social & Preventive Medicine, College of Medicine, Inha University, Incheon, Republic of Korea; 4 Department of Transdisciplinary Healthcare Sciences, Graduate School of Transdisciplinary Health Sciences, Yonsei University, Seoul, Republic of Korea; 5 Department of Epidemiology and Health Promotion, Institute for Health Promotion, Graduate School of Public Health, Yonsei University, Seoul, Republic of Korea; 6 Department of Big Data Service, National Health Insurance Service, Wonju, Republic of Korea; 7 Department of Health Administration, Yonsei University Graduate School, Wonju, Republic of Korea; 8 Health Insurance Policy Research Institute, National Health Insurance Service, Wonju, Republic of Korea; 9 Graduate School of Cancer Science and Policy, National Cancer Center, Goyang, Republic of Korea; 10 Division of Health Hazard Response, Korea Disease Control and Prevention Agency, Cheongju, Republic of Korea; 11 Department of Health Administration, College of Health Science, Dankook University, 119 Dandae-ro, Dongnam-gu, Cheonan-si, Chungcheongnam-do 31116 Republic of Korea; 12 Institute of Convergence Healthcare, Dankook University, Cheonan, Republic of Korea

**Keywords:** Korean National health examination baseline (KNHEB) cohort, Modifiable risk factors, Non-communicable diseases (NCDs), Tobacco control policy, Disease burden

## Abstract

**Background:**

The Korean National Health Examination Baseline (KNHEB) cohort was established in 2019 by the Korea Disease Control and Prevention Agency and the National Health Insurance Service to address research gaps and improve standardized monitoring of the health effects of smoking and other modifiable risk factors. It provides scientific evidence to inform national policies on tobacco control and other health determinants, aiming to reduce preventable mortality and disease burden in South Korea.

**Methods:**

The cohort includes 8,916,544 individuals aged ≥ 20 who underwent general health screenings in 2002–2003. It integrates three linked databases: insurance eligibility, medical visits (diagnostic codes, healthcare utilization), and health check-ups (behavioral risk factors, blood test results, etc.). Medical visit and health check-up data were collected until December 2018, while mortality records have been updated through 2019 and continue to be updated annually. At baseline, the mean age of participants was 44.2 years (SD 13.8). The mean follow-up duration was 16.2 years (SD 2.6) for health check-ups among all participants and 9.7 years (SD 4.6) for mortality among deceased individuals. The cohort enables long-term analysis of health outcomes, including cause-specific mortality based on death records and disease incidence identified through diagnostic codes and medical visit data.

**Findings to date:**

Analyses using the KNHEB cohort have provided key insights into smoking-related health risks. One study estimated that 60,213 smoking-attributable deaths occurred in South Korea in 2020, while another identified smoking intensity as the strongest predictor of all-cause mortality. Ongoing research include examining the effect of combined health-related factors (HRFs) on cause-specific mortality across age groups and investigating long-term smoking trajectories and alcohol consumption patterns in relation to major non-communicable diseases (NCDs).

**Conclusions:**

The KNHEB cohort provides a large-scale, population-based dataset that supports comprehensive analyses of the long-term effects of modifiable risk factors on NCDs. Its findings contribute to evidence-based policymaking in South Korea and offer comparative insights for global research on chronic disease prevention and risk factor management. Furthermore, its standardized data collection and integration with health records facilitate cross-country comparisons, reinforcing its value as a model for large-scale epidemiological studies on NCDs.

## Background

In 2019, non-communicable diseases (NCDs) accounted for 74% of global deaths (41 million people), making them the leading causes of mortality worldwide [1, 2]. South Korea exhibits mortality patterns similar to those of other high-income countries, including the United States, Germany, and Japan, where cancer and cardiovascular diseases are the primary causes of death [3–6]. However, unlike some Western nations where cardiovascular mortality has stabilized or declined, both cancer- and cardiovascular-related deaths continue to rise in South Korea. This increase is primarily driven by population aging, urbanization, and lifestyle changes, including physical inactivity, unhealthy diets, and increased exposure to environmental risk factors [7, 8]. These patterns highlight the need for ongoing surveillance and targeted interventions to address modifiable risk factors and reduce the burden of NCDs. Given these trends, international initiatives such as Sustainable Development Goal 3 of the 2030 Agenda for Sustainable Development emphasize the importance of reducing NCD-related mortality through prevention and treatment [9].

The key risk factors for NCDs include genetic, environmental, sociodemographic, and medical factors along with modifiable lifestyle factors such as smoking, alcohol consumption, physical inactivity, and obesity [10]. Among these, smoking and alcohol consumption remain significant public health concerns in South Korea. While smoking prevalence among male declined from 60.9% in 2001 to 32.4% in 2023, it remains high compared to other OECD countries [11]. In contrast, female smoking prevalence fluctuated from 5.2 to 6.3% over the same period. Monthly alcohol consumption decreased slightly among male (72.6% in 2005 to 68.0% in 2023) but increased among female (37.0–50.1%). High-risk drinking prevalence remained stable for male (19.9% in 2023), while for female, it more than doubled from 3.4% in 2005 to 7.7% in 2023 (Korea National Health and Nutrition Examination Survey, cited in KOSIS [12]). These behaviors significantly contribute to the disease burden in South Korea. In 2019, tobacco use was the leading risk factor for mortality, with a substantial impact on cancer (14.5% of total deaths) and cardiovascular diseases (4.8% of total deaths) in males. Alcohol consumption also remains a key contributor to morbidity and mortality, particularly through its association with digestive diseases and cancer [7]. Given the high burden of smoking- and alcohol-related diseases, strengthening evidence-based policies targeting these modifiable risk factors is essential for reducing the burden of NCDs and improving population health in South Korea.

Despite ongoing policy efforts, quantifying the long-term health effects of modifiable risk factors remains challenging due to delayed disease onset and evolving exposure patterns. Previous studies estimating smoking-attributable mortality and disease burden in South Korea have yielded inconsistent results, primarily due to small sample sizes, short follow-up durations, and methodological variability [13, 14]. Some studies have attempted to improve accuracy by integrating multiple data sources with varying tracking periods and characteristics [15, 16]. However, these studies still have limitations, such as restricted generalizability due to sampling constraints, short follow-up periods, and reliance on a single baseline assessment, which does not account for changes in smoking behavior over time. To address these challenges, a large-scale, population-based cohort with repeated exposure assessments is required to improve long-term estimates of smoking-related health impacts. Moreover, integrating multiple health data sources within a unified framework would enhance the ability to monitor risk factor trends and evaluate the effectiveness of public health policies.

The Korean National Health Examination Baseline (KNHEB) cohort was initially designed to develop a standardized framework for monitoring and evaluating the health effects of smoking in South Korea. Established in 2019 by the Korea Disease Control and Prevention Agency and National Health Insurance Service (NHIS), the cohort aimed to provide long-term, reliable estimates of smoking-attributable mortality and disease burden, with findings reported to the WHO Framework Convention on Tobacco Control (FCTC) [17]. However, its scope has since expanded beyond smoking-related research. Currently, the KNHEB cohort serves as a comprehensive platform for investigating the long-term effects of multiple modifiable risk factors for NCDs, including alcohol consumption, physical inactivity, and obesity. By integrating extensive health screening, medical utilization, and mortality records, the cohort enables a more precise assessment of risk factors and informs evidence-based interventions to reduce the NCD burden at both national and international levels.

## Methods

### Who is in the cohort?

Participants in the KNHEB were recruited from individuals who underwent general health screening in 2002 and/or 2003 under the National Health Insurance Act. Health insurance is mandatory for all residents of South Korea, and in 2000, the country adopted a single-payer health insurance system. The NHIS now serves as the sole public insurer, covering over 97% of the population, while the remaining 3% are covered under the Medical Aid program [18]. To facilitate early disease detection and ensure access to appropriate medical care, the National Health Screening Program was established under the National Health Insurance Act. In 2002, the program covered all health insurance subscribers, and their dependents aged ≥ 40 years [19]. The eligibility criteria were later expanded to include for dependents aged ≥ 20 years and to incorporate Medical Aid beneficiaries. The program is administered biennially by the NHIS, while manual laborers are required to undergo annual screenings. The screening participation rate increased from 43.2% in 2002 to 48.0% in 2003 and 74.1% in 2019 [20–22].

The NHIS consolidates nearly all medical utilization records in South Korea, except for non-reimbursed services and those covered by private insurance. This centralization enables the systematic collection, long-term storage, and structured management of health records, facilitating retrospective cohort studies, such as the KNHEB cohort [23]. Since 2012, the National Health Information database has provided claims data in two formats: sample research databases [24] and customized database [25], both consisting of de-identified individuals. This system was developed to support epidemiological and public health research by enabling structured access to administrative health records. Customized databases contain data extracted and processed according to specific research objectives and are provided by the Big Data Analysis Center. The KNHEB cohort data were retrieved from a customized database for national health-insurance records.

**Fig. 1:**
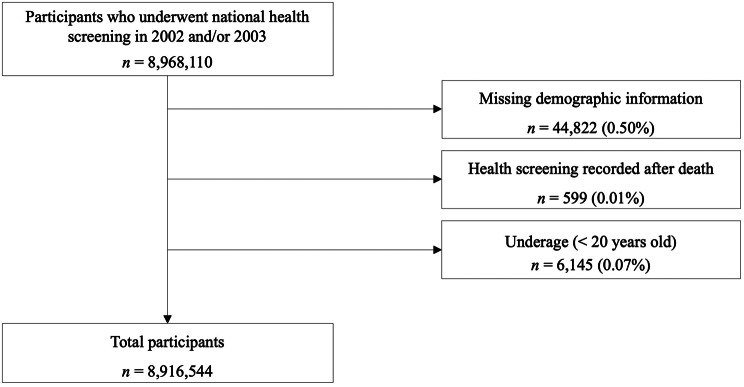
Flow chart of study participants

Among the 8,968,110 individuals who underwent general health screening in 2002 and/or 2003, the following were excluded: 44,822 individuals (0.50%) with incomplete demographic information (missing age or sex in insurance eligibility database), 6,145 individuals (0.07%) under 20 years old, and 599 individuals (0.01%) whose health screenings were recorded after their registered date of death. Since age and sex are fundamental demographic variables required for epidemiological analyses, and mortality is a key outcome, missing or inconsistent records could not be reliably corrected or imputed. Given the administrative nature of the dataset, these exclusions were necessary to maintain data accuracy and ensure cohort validity. The final cohort comprised 8,916,544 participants (Fig. 1).

Participants in the KNHEB cohort were retrospectively identified from individuals who participated in the national health screening program in 2002 and/or 2003. Since this study relies on legally authorized administrative data, individual consent was not required. The establishment of the cohort and the use of de-identified data were approved by the Institutional Review Board (IRB) of Seoul National University (Approval Number: E2104/002–013).

To assess the representativeness of the KNHEB cohort, Table 1 compares its sociodemographic characteristics with those of a nationally representative sample from the 2001 Korea National Health and Nutrition Examination Survey (KNHANES). KNHANES employs a complex, multi-stage probability sampling design to ensure representativeness of the non-institutionalized civilian population and serves as a national surveillance program for health status and behaviors in South Korea [26, 27]. Since the KNHEB cohort includes only adults, comparisons were made using the adult subset of KNHANES. Overall, the KNHEB cohort closely matches the general population in terms of sex, age, and health behavior distribution. Among KNHEB participants, 59.5% were male, 27.3% were in their 40s, and 22.9% were in their 30s, with no significant differences in age distribution compared to KNHANES. Smoking prevalence was also similar (KNHEB: 29.9%, KNHANES: 30.2%), as was alcohol consumption, with 47.4% of KNHEB participants reporting never drinking and 3.1% reporting daily alcohol consumption.

**Table 1:** General characteristics of KNHEB^*^ cohort participants (2002–2003) and KHANES^**^ participants (2001) (unit: %)

	KNHEB cohort, 2002 − 2003 (*n* = 8,916,544)	KNHANES cohort, 2001 (*n* = 27,318)
Sex		
Male	59.5	47.1
Female	40.5	52.9
Age (y)		
Mean (SD)	44.2 (13.8)	44.3 (0.2)
19 − 29	16.2	21.2
30 − 39	22.9	24.5
40 − 49	27.3	22.9
50 − 59	16.7	13.5
60 − 69	11.9	11.0
≥ 70	4.8	6.9
Insurance type		
Self-employed insured	24.0	47.2
Employed insured	76.0	49.0
Medical aid beneficiary	0.0	3.1
Risk factors		
Smoking status		
Never smokers	58.4	61.1
Former smokers	9.3	8.7
Current smokers	29.9	30.2
Drinking frequency		
Never	47.4	57.4
2 − 3 per month	20.8	14.2
1 − 2 per week	20.4	15.4
3 − 4 per week	6.6	7.3
Almost everyday	3.1	4.8
Physical activity		
Never	54.7	72.4
1 − 2 per week	25.7	6.9
3 − 4 per week	8.9	8.7
5 − 6 per week	2.2	7.0
Almost everyday	5.0	4.8
Weight status		
Underweight (BMI^***^ < 18.5)	4.1	5.4
Normal (18.5 ≤ BMI < 25)	64.5	64.5
Obese (BMI ≥ 25)	31.4	30.1

^*^ KNHEB: Korean National Health Examination Baseline

^**^ KHANES: Korea National Health and Nutrition Examinations Survey

^***^ BMI, body mass index

All risk factor variables were collected using the same methods in both datasets, except for physical activity. The KNHEB cohort used categorical response options for physical activity (as shown in the table), whereas KNHANES recorded open-ended numeric responses for weekly exercise frequency. While responses were categorized to align with the KNHEB format for comparison, differences in questionnaire structure may have influenced the distribution

The higher proportion of employed persons in the KNHEB cohort is due to the mandatory general health screening program, which is offered to individuals with employer health insurance. In the KNHEB cohort, 76% (including dependents) were employed subscribers, whereas in KNHANES, self-employed and employed subscribers were more evenly distributed. This difference is consistent with findings from the 2003 National Health Screening Report published by the NHIS, which indicated that individuals with employer-based insurance were more likely to participate in the national health screening program than those with self-employed insurance, likely due to differences in workplace health policies and access [28].

### How often have they been followed-up?

The cohort database includes various health-related data, such as insurance eligibility, death records, medical institutions visit history, and health check-up results.

Health check-up results were recorded whenever participants underwent a general health examination; however, no follow-up data were available for those who did not undergo subsequent examinations. The history of visits to medical institutions, including clinics, hospitals, tertiary hospitals, and public health centers, was recorded for inpatient and outpatient care. These records comprehensively capture medical utilization across different levels of the healthcare system in South Korea. Follow-up for health-examinations and medical institution visits continued until December 31, 2018. Death records were obtained through official death registration with Statistics Korea, which is legally required for all deaths in South Korea. These records were updated to 2019 and continue to be updated annually. The mean number of health check-ups per participant was 7.2 (SD 4.2), with a mean follow-up period of 16.2 years (SD 2.6) and a median follow-up of 17.1 years. By 2019, 840,491 participants had died, with a mean follow-up period until death of 9.7 years (SD 4.6) and a median of 10.2 years. Table 2 presents the biennial attrition rate of health screening participants, accounting for deaths and loss to follow-up over time. The health screening attrition rate was 16.3% over the 2002–2017 period.

**Table 2:** Health screening attrition rate by year

Screening Period^1)^	Number of participants, A^2)^	Number of deaths, B	Number of participants lost to follow-up, C	Attrition rate, C/A (%)
Baseline (2002–2003)	8,916,544	19,213	-	-
2004–2005	8,897,331	64,457	214,084	2.4
2006–2007	8,832,874	78,063	103,424	1.2
2008–2009	8,754,811	88,389	97,793	1.2
2010–2011	8,666,422	99,071	125,519	1.5
2012–2013	8,567,351	109,789	169,923	2.1
2014–2015	8,457,562	118,217	234,160	3.0
2016–2017	8,339,345	128,571	512,284	6.9
2002–2017	8,916,544	705,770	1,457,187	16.3

^1)^ Since the National Health Screening is conducted biennially, except for manual labourers, the attrition rate is calculated in two-year intervals.who

^2)^ The number of individuals eligible for follow-up, excluding those who died (B) or were lost to follow-up (C) in the previous period

### What has been measured?

The main variables in the databases, including temporal changes, are presented in Table 3. The insurance eligibility database includes basic demographic characteristics. The death database, obtained from Statistics Korea, includes the date and ICD-10 coded cause of death. In South Korea, all deaths must be legally registered with Statistics Korea under the Statistics Act and the Act on the Registration of Family Relations. Death registration data are supplemented with records from other administrative agencies to enhance accuracy and completeness. The accuracy rate of cause-of-death statistics is 91.9% [29], and the concordance between recorded and actual underlying causes of death is high when the cause is correctly specified on death certificates.

**Table 3:** Major variables in the Korean National health examination baseline (KNHEB) cohort database

Domain	Variables	2002	2003	2004	2005	2006	2007	2008	2009	2010	2011	2012	2013	2014	2015	2016	2017	2018
Health behaviours																		
Cigarette smoking	Smoking status	O	O	O	O	O	O	O	O	O	O	O	O	O	O	O	O	O
	Daily smoking amount for current smoker (categorical)	O	O	O	O	O	O	O										
	Daily smoking amount for current smoker (continuous)								O	O	O	O	O	O	O	O	O	O
	Smoking duration for current smoker (categorical)	O	O	O	O	O	O	O										
	Smoking duration for current smoker (continuous)								O	O	O	O	O	O	O	O	O	O
	Smoking dose for past smoker								O	O	O	O	O	O	O	O	O	O
	Smoking duration for past smoker								O	O	O	O	O	O	O	O	O	O
	Year smoking started				O	O	O	O										
	Year smoking ended					O	O	O										
Alcohol consumption	Frequency of alcohol consumption (categorial)	O	O	O	O	O	O	O										
	Amount of alcohol consumed (categorical)	O	O	O	O	O	O	O										
	Days of drinking per week								O	O	O	O	O	O	O	O	O	O
	Number of drinks per day								O	O	O	O	O	O	O	O	O	O
Physical activity	Days of physical activity per week (categorical)	O	O	O	O	O	O	O										
	Days of vigorous activity per week								O	O	O	O	O	O	O	O	O	O
	Days of moderate activity per week								O	O	O	O	O	O	O	O	O	O
	Days of walking per week								O	O	O	O	O	O	O	O	O	O
Obesity	Body mass index	O	O	O	O	O	O	O	O	O	O	O	O	O	O	O	O	O
	Waist circumstance							O	O	O	O	O	O	O	O	O	O	O
**Health problems**																		
Hypertension	Systolic blood pressure	O	O	O	O	O	O	O	O	O	O	O	O	O	O	O	O	O
	Diastolic blood pressure	O	O	O	O	O	O	O	O	O	O	O	O	O	O	O	O	O
Diabetes mellitus	HbA1C	O	O	O	O	O	O	O	O	O	O	O	O	O	O	O	O	O
	Fasting blood glucose	O	O	O	O	O	O	O	O	O	O	O	O	O	O	O	O	O
Dyslipidemia	Total cholesterol	O	O	O	O	O	O	O	O	O	O	O	O	O	O	O	O	O
	Triglycerides (TG)								O	O	O	O	O	O	O	O	O	O
	High-density lipoprotein (HDL)								O	O	O	O	O	O	O	O	O	O
	Low-density lipoprotein (LDL)								O	O	O	O	O	O	O	O	O	O
Kidney/urinary disease	Serum creatinine								O	O	O	O	O	O	O	O	O	O
	Glomerular filtration rate (GFR)								O			O	O	O	O	O	O	O
	Urine glucose	O	O	O	O	O	O	O										
	Urine protein																	
	Occult haematuria	O	O	O	O	O	O	O										
	Urine pH	O	O	O	O	O	O	O										
Liver disease	Aspartate aminotransferase (AST)	O	O	O	O	O	O	O	O	O	O	O	O	O	O	O	O	O
	Alanine aminotransferase (ALT)	O	O	O	O	O	O	O	O	O	O	O	O	O	O	O	O	O
	Gamma-glutamyl transpeptidase (ϒ-GTP)	O	O	O	O	O	O	O	O	O	O	O	O	O	O	O	O	O
	Antibody to hepatitis B	O	O	O	O	O	O	O										
Mental health	Cognitive impairment based on the Korean Dementia Screening Questionnaire-Cognition (KDSQ-C)	O	O	O	O	O	O	O										
	Stress (categorical)								O	O	O	O	O	O	O	O	O	O
Others	Trauma and aftereffect	O	O	O	O	O		O	O	O	O	O	O	O	O	O	O	O
Death	Vital statistics including dates and cause of deaths (ICD-10 codes)	O	O	O	O	O	O	O	O	O	O	O	O	O	O	O	O	O
**Medical history and family history**																		
Medical history	Presence of a condition (open question): liver disease, hypertension, stroke, heart disease, diabetes mellitus, cancer, etc.	O	O	O	O	O	O	O										
	Presence of a condition: stroke, heart disease (myocardial infarction/angina), hypertension, diabetes mellitus, dyslipidemia, pulmonary tuberculosis, etc.								O	O	O	O	O	O	O	O	O	O
	Year of onset	O	O	O	O	O	O	O										
	Full recovery or not	O	O	O	O	O	O	O										
	Drug treatment or not								O	O	O	O	O	O	O	O	O	O
Family history	Family history of disease: liver disease, cancer	O	O	O	O	O	O	O										
	Family history of disease: hypertension, stroke, heart disease, diabetes mellitus	O	O	O	O	O	O	O	O	O	O	O	O	O	O	O	O	O
	Family history of disease: others (including cancer)								O	O	O	O	O	O	O	O	O	O
**Hospital examination**																		
	Height	O	O	O	O	O	O	O	O	O	O	O	O	O	O	O	O	O
	Weight	O	O	O	O	O	O	O	O	O	O	O	O	O	O	O	O	O
	Visual acuity	O	O	O	O	O	O	O	O	O	O	O	O	O	O	O	O	O
	Auditory acuity	O	O	O	O	O	O	O	O	O	O	O	O	O	O	O	O	O
	Chest X-ray result	O	O	O	O	O	O	O	O	O	O	O	O	O	O	O	O	O
	EKG result	O	O	O	O	O	O	O										
**Health facility usage**																		
	Date of visit, type of medical institution (clinic/hospital/tertiary hospital/public health centre), type of visit (inpatient/outpatient/emergency/intensive care), length of stay, medical costs (insurer/patient), five diagnostic codes including main diagnosis and sub-diagnosis	O	O	O	O	O	O	O	O	O	O	O	O	O	O	O	O	O
**Socioeconomic and demographic factors**																		
	Sex, age, area of residence, insurance type, insurance contributions (proxy for income), type and grade of disability	O	O	O	O	O	O	O	O	O	O	O	O	O	O	O	O	O

The medical institution history database, maintained by the NHIS, is derived from administrative claims data. Each claim contains up to five diagnostic codes, including primary and sub-diagnosis, recorded using ICD-10 codes. These diagnostic codes serve as the basis for defining disease incidence in epidemiological studies, where operational definitions are applied. For instance, new cases may be identified based on multiple outpatient visits, at least one hospitalization with a relevant diagnostic code, or the initiation of disease-specific medication.

The health check-up database includes results from standardized health examinations and self-reported surveys. The Framework Act on Health Examinations mandates screenings to assess health status, prevent diseases, and enable early detection through clinical and laboratory tests as well as structured health surveys [19]. Under this mandate, the Ministry of Health and Welfare oversees quality management of the screening program, conducting regular re-evaluations of screening items to assess their validity. As part of these regulations, significant modifications to health examination components were introduced in 2008, including the expansion of laboratory tests and body measurements and the revision of questionnaires on smoking, alcohol consumption, and physical activity. For example, smoking and alcohol consumption variables were changed from categorical to continuous measures, allowing for the calculation of average intake levels.

## Findings to date

The KNHEB cohort was initially designed to establish a standardized framework for monitoring smoking-related mortality and disease burden in South Korea. One key achievement was the estimation of smoking-attributable mortality (SAM) based on 2019 data, which was reported to the WHO FCTC in 2023 [30, 31]. This analysis estimated that 58,036 deaths (male: 50,942; female: 7,094) were attributable to smoking, with lung cancer, stroke, ischemic heart disease, and diabetes being the leading causes of smoking-related deaths. The inclusion of these estimates in the FCTC report underscores the cohort’s role in informing national and global tobacco control strategies.

Building on this, a subsequent study using the KNHEB cohort —along with three other large Korean cohorts—provided a comprehensive estimate of SAM for 2020, which was published [32]. The study estimated that smoking caused 60,213 deaths in 2020, with lung cancer remaining the leading cause of mortality. The population-attributable fraction (PAF) was 33.2% in male and 4.6% in female. Comparisons with other countries illustrate regional differences in smoking-related mortality: Japan reported PAFs of 27.8% for males and 6.7% for females, while the United States reported 20.6% for males and 15.1% for females. The relatively high attributable fraction in Korean male aligns with the historically high smoking prevalence, whereas the lower PAF in Korean female likely reflects lower smoking rates compared to Western populations. These findings highlight the importance of continuous monitoring of smoking-related deaths and long-term tobacco control efforts.

Another study using the KNHEB cohort examined the association between various smoking indices and all-cause mortality risk [33]. This study assessed six smoking measures—ever-smoking status, smoking status (never, former, current), current smoking, smoking duration, smoking intensity (cigarettes per day), and pack-years—to determine the most informative predictor of mortality risk. Smoking intensity showed the strongest association with all-cause mortality in both sexes. Among male, pack-years provided the best explanation for smoking-related mortality, whereas in female, smoking intensity best explained mortality. However, smoking status also demonstrated reasona model fit and comparable effect sizes to those of smoking intensity in both sexes.

Expanding beyond smoking, ongoing research using the KNHEB cohort explores the impact of modifiable risk factors on cause-specific mortality, focusing on their combined effects rather than isolated influences Studies suggest that analyzing individual risk factors separately may oversimplify complex biological interactions, whereas integrating multiple health-related factors (HRFs) into a composite score improves predictive accuracy and better captures population-level variations [34]. This approach enables a more comprehensive assessment of mortality risk, offering valuable insights for targeted prevention strategies.

The KNHEB cohort remains a key resource for investigating modifiable risk factors and their impact on NCDs. Ongoing research explores long-term smoking patterns using trajectory modeling to assess risks across different age groups. Additionally, studies are underway to compare survival rates between cancer patients who quit smoking after diagnosis and those who continue smoking. Future research will further examine the role of modifiable risk factors across a broader range of NCDs. Utilizing the cohort’s extensive health screening, medical utilization, and mortality records, these studies will provide critical insights for disease prevention and health policy in South Korea.

## Strengths and weaknesses

The KNHEB cohort, consisting of approximately 8.9 million participants with two decades of follow-up, has several strengths. First, the cohort is established within South Korea’s single-payer health insurance system, enabling linkage with national health databases, including medical claims, death records, and national health screenings. These integrated data sources support long-term surveillance, healthcare utilization tracking, and outcome assessment with minimal loss to follow-up. Second, the cohort benefits from a legally mandated national health screening program, ensuring standardized and periodic health assessments. While screening components may change with policy updates, the program’s structured framework faciliatates consistent data collection on biological markers, anthropometric measures, and behavioral risk factors. Third, the KNHEB cohort combines health screening data with real-world healthcare utilization records, allowing for an objective assessment of modifiable risk factors and chronic disease progression. Unlike studies relying solely on self-reported data, it includes prescription records, hospital visits, and cause-specific mortality, supporting a comprehensive evaluation of disease onset, progression, and treatment patterns.

The KNHEB cohort study also has limitations. Participants in the cohort underwent health screenings, which may introduce selection bias toward individuals capable of participating in such screenings. Despite this, the demographic characteristics of the KNHEB cohort are similar to those of the KNHANES cohort, which is representative of the South Korean population. Another limitation is the reliance on claims data, where diagnostic codes are recorded for billing rather than research. Operational definitions using repeated visits, hospitalizations, or prescriptions help improve accuracy, but a short prevalence period may misclassify pre-existing conditions as new cases, inflating incidence—especially for chronic diseases like diabetes [35]. Additionally, temporary spikes in blood glucose or blood pressure can lead to coding without a confirmed diagnosis, and suspected cases may be misclassified as confirmed. Incorporating repeated visits or prescriptions can help mitigate these issues. Despite these limitations, cancer incidence estimates from claims data closely align with national registry records, supporting their validity for epidemiologic research [36].

## Conclusion

The KNHEB cohort provides a large-scale, population-based resource for studying the long-term effects of modifiable risk factors on health. With extensive follow-up and integration of health screening, medical utilization, and mortality records, it enables comprehensive assessments of NCD risks. Recent findings have advanced understanding of smoking-related mortality and expanded research to broader modifiable risk factors, including alcohol consumption and combined health-related factors, improving insights into lifestyle-related disease burdens and cause-specific mortality. These findings complement Western-based studies, offering valuable perspectives from an East Asian population. Structured within a national health insurance system and a legally mandated health screening program, the cohort ensures standardized, repeated assessments, making it a reliable tool for tracking disease risk and evaluating prevention strategies. Its design serves as a model for leveraging administrative and clinical data in large-scale epidemiological research. Beyond South Korea, findings from this cohort can contribute to global efforts to refine risk assessment models and tailor prevention strategies. As research progresses, the dataset will continue to support evidence-based health policies and enhance understanding of lifestyle-related disease burdens worldwide.

## Data Availability

The KNHEB data can be accessed through the Health Insurance Data Service website (http://nhiss.nhis.or.kr). Researchers must obtain approval from their institutional review board before submitting a research proposal, which is subject to review and approval by the Review Committee of the National Health Insurance Service before data access is granted.

## Abbreviations

KNHEB: Korean National Health Examination Baseline
NCDs: Non-Communicable Diseases
NHIS: National Health Insurance Service
FCTC: Framework Convention on Tobacco Control
KNHANES: Korea National Health and Nutrition Examination Survey
SD: Standard deviation
ICD-10: International Classification of Diseases, 10th Revision
SAM: Smoking-Attributable Mortality
HRFs: Health-related factors
